# Correction to: “LCoRL Regulates Growth and Metabolism”

**DOI:** 10.1210/endocr/bqaf175

**Published:** 2025-12-19

In above-named article by Wyler SC, Gahlot S, Bideyan L, Yip C, Dushime J, Chen B, Lee JJ, Tinajero A, Limboy C, Bordash S, Heaselgrave SR, Nguyen T-N, Lee S, Bookout A, Lantier L, Fowlkes JL, You Y-J, Fujikawa T, and Elmquist JK (*Endocrinology*, 2024, 165(12); doi: 10.1210/endocr/bqae146) there were errors in the authorship roster.

In the original article, Louise Lantier’s name was incorrectly published as “Loise Lantier.” In the updated article, Louise Lantier’s name has been listed correctly.

The article has been corrected online.

doi: 10.1210/endocr/bqae146

